# Reflectance confocal microscopy in the management of lentigo maligna and lentigo maligna melanoma: a systematic review

**DOI:** 10.1016/j.jpra.2026.02.022

**Published:** 2026-03-04

**Authors:** Olivier Mathieu, Douglas Henderson, Emilien Ezine, Kevin Serror, Barouyr Baroudjian, Maxime Battistella, Céleste Lebbé, Marc Chaouat, David Boccara

**Affiliations:** a Department of Plastic and Reconstructive Surgery, Hôpital Saint-Louis, Assistance Publique – Hôpitaux de Paris, Université Paris Cité, Paris, France; b Department of Otolaryngology-Head Neck Surgery, Lariboisière Hospital, Assistance Publique – Hôpitaux de Paris, Université Paris Cité, Paris, France; c Department of Dermato-Oncology, Hôpital Saint-Louis, Assistance Publique – Hôpitaux de Paris, Université Paris Cité, Paris, France; d Department of Pathology, Hôpital Saint-Louis, Assistance Publique – Hôpitaux de Paris, Université Paris Cité, Paris, France; e Dermato-Oncology and CIC, Cancer Institute, Assistance Publique Hôpitaux de Paris, CNRS EMR80000, Paris, France; f INSERM U1342, Institut de Recherche Saint-Louis, Paris, France

**Keywords:** Lentigo maligna, Lentigo maligna melanoma, Margin mapping, Melanoma in situ, Non-invasive imaging, Reflectance confocal microscopy

## Abstract

**Background:**

Lentigo maligna (LM) and lentigo maligna melanoma (LMM) are difficult to manage because of their subclinical extension and ill-defined margins, especially on chronically sun-damaged facial skin. Reflectance confocal microscopy (RCM), a non-invasive imaging technique with near-histological resolution, has emerged as a valuable adjunct for diagnosis, presurgical margin mapping, and postoperative surveillance.

**Objective:**

To systematically review current evidence on the role of RCM in LM and LMM management.

**Methods:**

A systematic review was performed in accordance with PRISMA 2020 guidelines. PubMed, Embase, and Scopus were searched up to July 2025. Eligible studies assessed RCM for diagnostic accuracy, presurgical mapping, surgical outcomes, or follow-up. Findings were synthesized qualitatively.

**Results:**

Twenty-nine studies were included. Across diverse study designs, RCM demonstrated higher diagnostic accuracy than dermoscopy for LM, with reported sensitivities ranging from 85 % to 100 % and specificities from 71 % to 97 %. In comparative studies and meta-analyses, RCM showed superior specificity compared with dermoscopy and improved discrimination between LM and early invasive LMM. Presurgical RCM margin mapping was associated with higher first-stage clearance rates (up to 63.6 % vs. 34.7 % with standard assessment), fewer surgical stages (mean 1.1–1.2 vs. 1.7–1.9), narrower excision margins, and lower local recurrence rates (approximately 2–5 % vs. up to 13 % in non-RCM series). RCM was also effective for monitoring non-surgical therapies and detecting early recurrences within surgical scars. Major barriers to widespread adoption include cost, limited availability, training requirements, and the absence of standardized protocols.

**Conclusions:**

RCM strengthens the management of LM and LMM by improving diagnostic confidence, enabling precise margin mapping, and supporting long-term surveillance. Broader adoption will depend on standardization of mapping techniques, integration into clinical workflows, and validation of artificial intelligence–assisted interpretation.

## Introduction

Lentigo maligna (LM) is a melanoma in situ arising on chronically sun-damaged skin of the head and neck in older adults, with a steadily increasing incidence. Its slow growth and ill-defined, subclinical extension make diagnosis and margin delineation challenging.^1^, ^2^, ^3^, ^4^, ^5^ If left untreated or incompletely excised, LM may progress to lentigo maligna melanoma (LMM), an invasive and life-threatening malignanc;y^6^^,^^7^ LMM develops in up to 3.5 % of LM cases each year.^8^

Because LM develops on chronically sun‐damaged skin, its margins often blend imperceptibly with adjacent solar lentigines, further challenging visual demarcation.^9^ Moreover, both clinical and dermoscopic assessment may underestimate the true extent of LM, leading to incomplete excision and high recurrence rates.^10^, ^11^, ^12^ Traditional wide local excision, while effective, often requires broad surgical margins, which can be problematic in facial locations.^13^ In this context, non-invasive imaging techniques such as reflectance confocal microscopy (RCM) have emerged as valuable adjuncts to clinical decision-making.

Reflectance confocal microscopy enables real-time, in vivo imaging of the skin at near-histological resolution, reaching depths of 200–300 µm sufficient to visualize most melanocytic proliferations.^14^ Two main devices are used (arm-mounted and handheld systems), offering complementary trade-offs between field size and flexibility.^15^ By providing horizontal optical sections, RCM highlights architectural disarray, cytologic atypia, and pagetoid spread on chronically sun-damaged skin.^16^ These characteristics make RCM particularly suited to LM/LMM, where subclinical extension and ill-defined margins challenge traditional assessment.

Despite growing adoption in specialized centers, RCM remains underutilized in routine practice due to cost, limited access, and training requirements. To date, no systematic review has comprehensively synthesized evidence across the entire clinical pathway of LM/LMM management. The present review was therefore designed to integrate available data on diagnostic performance, presurgical planning, surgical outcomes, non-surgical treatment monitoring, and postoperative surveillance, providing a unified and clinically oriented overview of the role of RCM throughout patient care.

## Methods

This systematic review was conducted in accordance with the Preferred Reporting Items for Systematic Reviews and Meta-Analyses (PRISMA) 2020 guidelines.^17^ The review protocol was registered with PROSPERO in September 2025 (registration number CRD420251147002), after completion of the initial literature search conducted in July 2025. No changes to eligibility criteria occurred after registration, and heterogeneity precluded meta-analysis. The completed PRISMA checklist is available in the Supplementary Material (Table 1).

**Table 1:** MINORS checklist summary for included studies.

Study	Item 1	Item 2	Item 3	Item 4	Item 5	Item 6	Item 7	Item 8	Item 9	Item 10	Item 11	Item 12	Total score
Guitera *et al*.^27^ (2010)	2	2	0	2	1	2	2	0	2	2	2	2	17/24
Guitera et al.^33^ (2013)	2	2	2	2	1	2	2	0	2	2	2	2	21/24
Guitera et al.^42^ (2014)	2	2	0	2	1	2	2	0	2	2	2	2	17/24
Champin et al.^34^ (2014)	2	2	2	2	1	2	2	0	2	2	2	2	21/24
Alarcon et al.^43^ (2014)	2	2	2	2	1	2	2	0	2	2	2	2	21/24
Menge et al.^28^ (2016)	2	2	2	2	1	2	2	0	2	2	2	2	21/24
Gómez-Martín et al.^25^ (2017)	2	2	2	2	1	2	2	0	2	2	2	2	21/24
Wurm et al.^65^ (2017)	2	2	2	2	1	2	2	0	2	2	2	2	21/24
Yélamos et al.^53^ (2017)	2	2	2	2	1	2	2	0	2	2	2	2	21/24
Couty et al.^35^ (2018)	2	2	2	2	1	2	2	0	2	2	2	2	21/24
Cinotti et al.^66^ (2018)	2	2	0	2	1	2	2	0	2	2	2	2	17/24
Cinotti et al.^40^ (2020)	2	2	2	2	1	2	2	0	2	2	2	2	21/24
Navarrete-Dechent et al.^44^ (2020)	2	2	0	2	1	2	2	0	NA	NA	NA	NA	11/16
Navarrete-Dechent et al.^67^ (2020)	2	2	0	2	1	2	2	0	NA	NA	NA	NA	11/16
Collgros et al.^32^ (2021)	2	2	0	2	1	2	2	0	NA	NA	NA	NA	11/16
Gao et al.^41^ (2021)	2	2	0	2	1	2	2	0	2	2	2	2	17/24
Elshot et al.^52^ (2021)	2	2	2	2	1	2	2	NA	NA	NA	NA	NA	13/16
Gouveia et al.^7^ (2023)	2	2	2	2	1	0	0	0	2	2	2	2	17/24
Guida et al.^68^ (2023)	2	1	0	2	1	0	0	0	2	2	2	2	14/24
Licata et al.^26^ (2023)	2	2	0	2	1	0	0	0	2	2	2	2	15/24
Navarrete-Dechent et al.^51^ (2023)	2	2	2	2	2	1	2	0	2	2	2	2	19/24
Cabrioli et al.^39^ (2023)	2	1	0	2	1	0	0	0	2	2	2	2	17/24
Stevens et al.^31^ (2024)	2	2	2	2	2	0	0	0	2	2	2	2	18/24
Pizzichetta et al.^69^ (2024)	2	2	0	2	1	0	0	0	2	2	2	2	15/24
Mathieu et al.^37^ (2025)	2	2	0	2	1	2	2	0	2	2	2	2	19/24

### Search strategy

A comprehensive search of PubMed, Embase and Scopus was performed from January 1, 2000 to July 1, 2025. The search strategy combined the following keywords with Boolean operators: *“lentigo maligna”, “lentigo maligna melanoma”, “reflectance confocal microscopy”, “RCM”, “VivaScope”, “handheld confocal”, “ex vivo confocal”, “margin mapping”*, and *“preoperative delineation”*. Searches were limited to English-language publications. In addition, one study authored by our group was manually added, as it was not yet indexed at the time of the search. This study corresponds to reference Mathieu et al.^37^

### Eligibility criteria

We included prospective and retrospective studies, case series, and systematic reviews or meta-analyses specifically evaluating RCM in LM or LMM. Eligible outcomes included diagnostic performance, presurgical margin delineation, surgical outcomes, and post-treatment surveillance.

We excluded narrative reviews without quantitative data, studies with no LM/LMM-specific analysis, conference abstracts without full-text publication, and non-relevant or non-English reports. Case reports with <10 patients were excluded unless they provided unique methodological insights.

### Study selection

**Figure 1:**
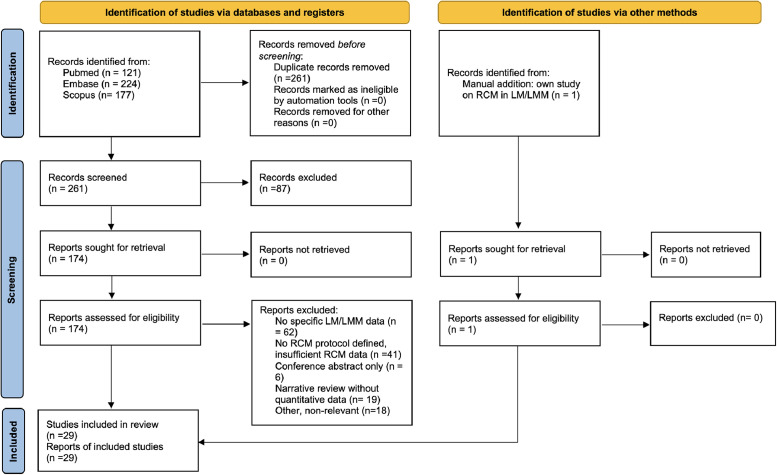
Flow diagram of study selection according to PRISMA 2020 guidelines.

Two reviewers (OM and DH) independently screened titles, abstracts, and full texts. Inter-reviewer agreement for study selection was assessed using Cohen’s kappa coefficient. Discrepancies were resolved through discussion and consensus, with involvement of a third senior author when necessary. The reasons for exclusion were recorded and are reported in the PRISMA flow diagram (Figure 1). Data were extracted into a standardized spreadsheet, including author, year, country, study design, sample size, lesion characteristics, type of RCM device and protocol, primary outcomes and follow-up.

### Quality assessment

The methodological quality of non-randomized studies was assessed using the Methodological Index for Non-Randomized Studies (MINORS)^18^ (Supplementary material Table 2), while systematic reviews and meta-analyses were appraised with the AMSTAR-2 tool^19^ (Supplementary material Table 3). Results are summarized in Table 1, Table 2.

**Table 2:** AMSTAR 2 checklist summary for included reviews.

	Nie (2021)	Elshot (2023)	Mesquita (2024)	Melhoranse Gouveia (2024)
PICO defined	Yes	Yes	Yes	Yes
Protocol registered	No	No	Yes (PROSPERO)	Yes (PROSPERO)
Study designs explained	Yes	Yes	Yes	Yes
Search comprehensive	Yes	Yes	Yes	Yes (8 DB)
Selection duplicate	No	Yes	Yes	Yes
Extraction duplicate	No	Yes	Yes	Yes
Excluded studies list	No	Partial	Yes	Yes
Included studies described	Yes	Yes	Yes	Yes
Risk of bias assessed	Yes (QUIPS)	Partial	Yes (QUADAS-2)	Yes
Funding reported	No	No	No	No
Meta-analysis appropriate	Partial	Yes	Yes	Yes
ROB impact assessed	Partial	Partial	Yes	Yes
Heterogeneity explained	Partial	Yes	Yes	Yes
Publication bias assessed	Yes	No	Yes	Yes
Limitations discussed	Yes	Yes	Yes	Yes
COI declared	Yes	Yes	Yes	Yes
Overall rating	Critically low	Moderate	High	High

## Results

The literature search resulted in a total of *n* = 522 studies, of which *n* = 29 were included (Figure 1). Inter-reviewer agreement for study selection was substantial (κ = 0.78). Methodological quality of the non-randomized studies, assessed using the MINORS checklist, ranged from 14 to 21 out of 24 for comparative studies and from 11 to 13 out of 16 for non-comparative studies, reflecting overall moderate to high quality. Systematic reviews were appraised using the AMSTAR-2 tool, with ratings ranging from critically low to high quality. Key study characteristics and impacts on management are summarized in Table 3.

**Table 3:** Key studies on RCM in LM and LMM.

Study	Year	Design (*n*=)	RCM type	Purpose	Key finding	Impact on management
Guitera et al.^27^	2010	Retrospective (*n* = 81 LM, 203 benign)	VivaScope 1500®	Differentiate LM from benign macules	LM score ≥ 2 → Sensitivity 85 %, Specificity 76 %	RCM improves diagnostic accuracy, reduced unnecessary biopsies
Guitera et al.^33^	2013	Prospective (*n* = 37 high-risk lesions)	VivaScope 1500®	Map subclinical extension	RCM prompted wider excision in 73 % cases	RCM prevents incomplete excision
Guitera et al.^42^	2014	Retrospective (*n* = 98 treated LM patients, 31 biopsied)	VivaScope 1500®	Detect recurrence after non-surgical treatment (imiquimod, RT, cryo)	LM score (RCM) sensitivity 100 %, specificity 94 %; dermoscopy sensitivity 80 %, specificity 56 %	RCM offers superior accuracy over dermoscopy for detecting LM recurrence, guiding timely biopsy and reducing missed failures
Champin et al.^34^	2014	Prospective (*n* = 33 LM)	VivaScope3000®	‘Spaghetti’ margin mapping	85 % first-pass clearance, 0 recurrences at 10 months	RCM reduces number of stages
Alarcon et al.^43^	2014	Prospective (*n* = 20 patients)	VivaScope1500®	Evaluate RCM for monitoring LM response to imiquimod when surgery is contraindicated	RCM identified 70 % responders with no false negatives	RCM enables non-invasive monitoring of LM treatment response, reducing need for repeated biopsies
Menge et al.^28^	2016	Prospective (*n* = 63 lesions)	VivaScope3000®	Real-time diagnostic scoring	Sensitivity 100 %, Specificity 71 %	High accuracy for equivocal lesions
Gomez-Martin et al.^25^	2017	Prospective (*n* = 61 lesions)	VivaScope 1500®	Assess diagnostic accuracy of RCM for LM compared to dermoscopy	Sensitivity 91.7 %, specificity 86.8 %	RCM improves diagnostic accuracy for LM compared with dermoscopy
Wurm et al.^65^	2017	Prospective (*n* = 70 lesions)	VivaScope1500®	Assess added value of RCM over dermoscopy for flat pigmented facial lesions	RCM sensitivity 95 %, specificity 82 %	RCM helps select lesions for biopsy, reduces unnecessary excisions
Yélamos et al.^53^	2017	Prospective (*n* = 23 LM/LMM analyzed)	VivaScope3000® (Handheld + videomosaicking)	Pre-surgical LM/LMM margin mapping	43 % quadrants with LM beyond dermoscopy margins; margins 0.76 mm smaller vs. histology	RCM-RV correlates well with histology, improves margin delineation, spares healthy tissue
Couty et al..^35^	2018	Prospective (*n* = 70 cases)	VivaScope3000®	Pre-op mapping	Avg 1.13 strips, 0 recurrences at 44 months	RCM improves margin control
Cinotti et al.^66^	2018	Multicenter retrospective (*n* = 223 lesions)	VivaScope3000®	Compare diagnostic accuracy of dermoscopy vs. RCM for LM/LMM	RCM was more sensitive (80 %, vs. 61 %) and less specific (81 % vs. 92 %) than dermoscopy for LM/LMM	RCM improves differentiation of LM/LMM from benign macules
Cinotti et al.^40^	2020	Prospective (*n* = 42 LM/LMM)	VivaScope3000® and VivaScope 2500®	Combine in vivo RCM + *ex vivo*	97.6 % correct margin classification	Combining RCM and EVCM provides accurate margin evaluation, supporting a new surgical strategy
Navarrete-Dechent et al.^44^	2020	Retrospective cohort (*n* = 29 patients)	VivaScope3000®	Differentiate true melanoma recurrence from benign repigmentation in LM/LMM scars	Sensitivity 95.2 %, specificity 77.7 %, PPV 90.9 %, NPV 87.5 %	RCM improves post-surgical surveillance, reducing unnecessary biopsies for benign pigmentation
Navarrete-Dechent et al.^67^	2020	Retrospective + prospective cohort (*n* = 67)	VivaScope 3000®	Evaluate incompletely excised LM/LMM	Accuracy 78.6 %, Sensitivity 90.9 %, Specificity 33.3 %, PPV 83.3 %	RCM helps detect residual tumor in incomplete excisions, improving surgical decision-making
Nie et al.^29^	2021	Meta-analysis (479 patients, 6 studies)	-	Compare RCM vs. dermoscopy	RCM higher specificity (MD 19.10 %, *p* = 0.04)	Better diagnostic precision than dermoscopy
Collgros et al.^32^	2021	Retrospective (*n* = 117 lesions)	-	Pre-op mapping	Recurrence 27 % vs. 38 % (RCM vs. standard)	RCM reduces recurrence
Gao et al.^41^	2021	Retrospective (*n* = 47 LM)	Handheld	Pre-op mapping	Repair time reduced (mean 14.6 days mapped vs. 27.0 nonmapped)	Faster reconstruction
Elshot et al.^52^	2021	Retrospective (*n* = 26 lesions)	VivaScope3000®	Pre-op mapping	90 % sensitivity, 86 % specificity, 4.8 % recurrence	Better margin clearance
Gouveia et al.^7^	2023	Prospective case-control (*n* = 229 lesions)	VivaScope 1500® and VivaScope3000®	To assess the accuracy of RCM for the detection of invasion component on LM/LMM lesions	89 % sensitivity for LMM detection; three key features (junctional disarray, large melanocytes, nests) predicted invasion (AUC 74 %)	RCM enables early identification of invasive component in LM, guiding surgical planning and avoiding undertreatment
Guida et al.^68^	2023	Retrospective multicenter (*n* = 180 lesions)	VivaScope 1500® and VivaScope3000®	Validate dermoscopic predictors for LM/LMM, identify RCM patterns, and assess correlations	LMM associated with medusa head-like structures, dermal nests, and nucleated papillary cells on RCM; strong correlation with dermoscopic features	Supports integrated RCM + dermoscopy use
Licata et al.^26^	2023	Retrospective multicenter (*n* = 48 EF-LM, 45 controls)	VivaScope 1500®	Diagnose extra-facial LM	Sensitivity 90 %, Specificity 97 %	Supports use of RCM for accurate diagnosis of EF-LM
Navarrete-Dechent et al.^51^	2023	Prospective (*n* = 72 patients)	VivaScope 3000®	Pre-op mapping	Sensitivity for residual melanoma 96.7 %, specificity 66.7 %; margin agreement with histology 85.9 % (κ = 0.71)	High histology concordance
Cabrioli et al.^39^	2023	Retrospective (*n* = 57 LM/LMM cases)	VivaScope 3000®	RCM vs. dermoscopy for margin mapping	sensitivity of 94.7 %, specificity of 83.3 %, positive predicted value 94.7 %, and negative predicted value 83.3 %	RCM significantly reduces need for re-intervention and overtreatment, particularly in cosmetically sensitive facial areas
Elshot et al.^10^	2023	Systematic review (5059 LM, 1271 LMM)	VivaScope 1500® and VivaScope3000®	Summarize mapping performance	Stages reduced (1.7→1.1)	Evidence for RCM mapping benefit
Stevens et al.^31^	2024	Prospective UK study (*n* = 734 lesions, 86 LM/MM)	VivaScope 1500® and VivaScope3000®	Assess diagnostic performance of RCM vs. clinical exam and dermoscopy for LM and MM in a UK cohort	RCM: sensitivity 94.2 %, specificity 83.2 %, NPV 99.1 %	Demonstrates RCM as a highly accurate adjunct for LM/MM diagnosis, reducing unnecessary biopsies and improving triage
Mesquita et al.^38^	2024	Meta-analysis (9 cohorts, *n* = 329)	VivaScope 1500® and VivaScope3000®	Summarize mapping performance	Sensitivity 91.4 %, Specificity 95.7 %, Avg stages 1.16	Strong support for RCM margin mapping
Melhoranse Gouveia *et al*.^23^	2024	Systematic review & pooled analysis (27 studies, 303 lesions)	VivaScope 1500® and VivaScope3000®	Identify RCM features predictive of early vs. advanced LM/LMM stages	LM: non-edged papillae (OR 4.50), widespread pagetoid and junctional atypical cells (OR 25.06), junctional nests (OR 18.06); LMM: epidermal & junctional disarray, destroyed collagen, dermal nests	Provides evidence-based RCM criteria for staging LM spectrum, improving diagnostic accuracy and guiding appropriate surgical margins
Pizzichetta et al.^69^	2024	Multicentre retrospective (*n* = 224 lesions; 55 AHLM/LMM, 62 AHBCC/AHSCC, 56 AHBL, 51 AK)	VivaScope 3000®	Evaluate diagnostic performance of dermoscopy, RCM, and their combination in AHLM/LMM	Dermoscopy & RCM >97 % accuracy vs. AHBCC/AHSCC; combo improved accuracy (98.0→99.1 %). For AHLM/LMM vs. benign/AK, accuracy modestly improved but high-confidence diagnoses increased (36.2→76.6 % vs. AHBLs)	Dermoscopy and RCM combination enhances diagnostic certainty for AHLM/LMM, reducing equivocal biopsies
Mathieu et al.^37^	2025	Retrospective cohort (*n* = 282; 184 RCM, 98 control)	VivaScope 3000®	Compare surgical outcomes with vs. without RCM presurgical mapping	Higher complete excision at first stage (63.6 % vs. 34.7 %, *p* < 0.0001); fewer stages (mean 1.18 vs. 1.94, *p* < 0.0001); narrower margins (5.2 vs. 6.2 mm, *p* = 0.002); benefit greatest in complex facial regions	RCM improves surgical efficiency and precision, supporting integration into routine preoperative planning

Abbreviations: LM = Lentigo Maligna, LMM = Lentigo Maligna Melanoma, RCM = Reflectance Confocal Microscopy, AHLM/LMM = Atypical Hyperpigmented Lentigo, AHBCC = Atypical Hyperpigmented Basal Cell Carcinoma, AHSCC = Atypical Hyperpigmented Squamous Cell Carcinoma, AHBL = Atypical Hyperpigmented Benign Lesions, AK = Actinic Keratosis.

### Diagnostic performance of RCM in LM and LMM

Reflectance confocal microscopy has emerged as a powerful non-invasive imaging tool that enhances diagnostic precision for LM and enables early detection of dermal invasion in LMM.

**Figure 2:**
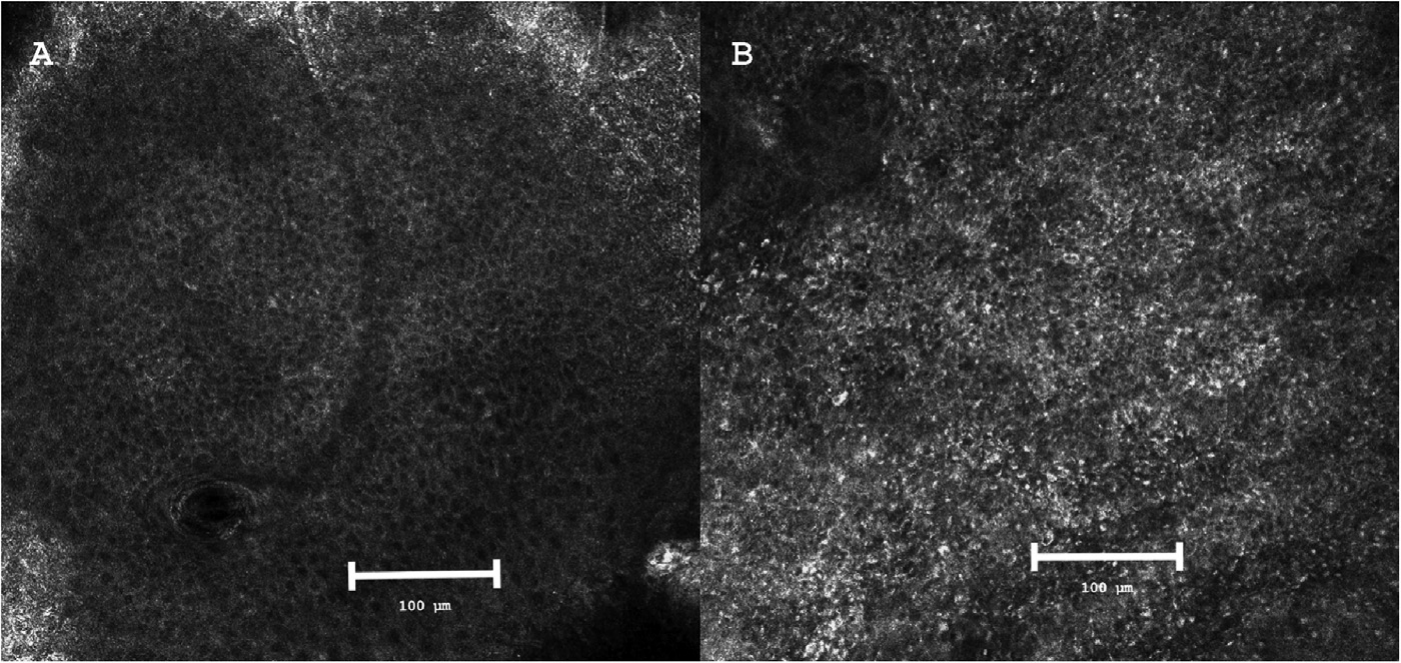
Reflectance confocal microscopy of normal skin versus lentigo maligna. (A) Normal epidermis showing a regular honeycomb pattern with preserved architecture. (B) Lentigo maligna with complete loss of the honeycomb structure, marked architectural disorganisation, and numerous hyperreflective round and dendritic cells consistent with severe atypia. Images adapted from Mathieu et al.^37^ and shown for illustrative purposes to depict characteristic and widely reported RCM features of normal skin and lentigo maligna.

Despite an established consensus glossary,^20^ terminology varies across LM/LMM studies.^21^^,^^22^ However, most analyses converge on three core dimensions of confocal morphology: architecture, cellular distribution and cell type. In normal skin, RCM shows a regular honeycomb pattern of polygonal keratinocytes with dark nuclei and bright cytoplasm and borders. In contrast, LM/LMM is characterized by architectural disorganization, the presence of atypical dendritic cells, and large round hyperreflective cells, correlating with histopathologic criteria of malignancy.^21^^,^^23^, ^24^, ^25^, ^26^ Representative RCM images illustrate the contrast between normal skin, showing a regular honeycomb pattern, and lentigo maligna, characterized by complete loss of epidermal architecture and the presence of atypical hyperreflective cells (Figure 2). From a clinical standpoint, several studies suggest that these RCM features help inform management decisions. The presence of non-edged dermal papillae and atypical dendritic or round pagetoid cells is commonly associated with a higher likelihood of LM and may guide targeted biopsy or complete excision. Conversely, extensive pagetoid spread and marked junctional disarray have been reported more frequently in invasive disease and may prompt closer histopathologic correlation or wider surgical margins, whereas their absence may support more conservative or tissue-sparing approaches. Across diagnostic studies, reported sensitivity and specificity of RCM for LM/LMM range broadly, reflecting substantial heterogeneity in study design, lesion subtype, and clinical context (Table 4). While high sensitivity is consistently observed, specificity and predictive values vary depending on factors such as actinic background skin, reader experience, and the indication for RCM (primary diagnosis, recurrent lesions, or invasion assessment).

**Table 4:** Diagnostic performance of reflectance confocal microscopy (RCM) in LM/LMM – comparative metrics with contextual interpretation.

Study	Population/design (n)	Reference standard	Sensitivity ( %)	Specificity ( %)	PPV ( %)	NPV ( %)	Important nuances/subgroup findings
Guitera et al.,^27^ 2010	Retrospective; 81 LM / 203 benign facial macules (+ validation set 29 LM / 44 benign)	Histopathology	85 (93 validation)	76 (82 validation)	NR	NR	LM score algorithm; good interobserver reproducibility (87 %); improved performance in validation cohort.
Menge et al.,^28^ 2016	Prospective; 63 equivocal lesions	Histopathology	100	71	85	100	Excellent sensitivity; false positives mainly due to actinic damage and benign mimickers.
Gómez-Martín et al.,^25^ 2017	Prospective; 63 ambiguous facial macules	Histopathology	91.7	86.8	NR	NR	False positives linked to basal melanocyte hyperplasia and Langerhans cells; detailed histopathologic correlation.
Wurm et al.,^65^ 2017	Prospective; 70 flat facial lesions	Histopathology	95	82	NR	NR	Sensitivity comparable to dermoscopy; risk of AK overdiagnosis without clinical correlation.
Cinotti et al.,^66^ 2018	Multicenter multi-reader study; 223 facial lesions total, including 115 LM/LMM (92 LM, 23 LMM)	Histopathology	80 (mean)	81 (mean)	NR	NR	Higher sensitivity for hypomelanotic and recurrent LM/LMM; improved interobserver agreement and diagnostic confidence; sROC AUC ≈0.89.
Navarrete-Dechent et al.*,*^44^ 2020	Retrospective; 30 scar lesions (29 patients)	Histopathology	95.2	77.7	90.9	87.5	Evaluation of repigmentation after surgery; LM-specific dermoscopic criteria frequently absent.
Licata et al.*,*^26^ 2023	Multicenter case–control; 48 extra-facial LM / 45 controls	Histopathology	90	97	NR	NR	Focused on extra-facial LM; distinct dermoscopic and RCM patterns compared to facial LM.
Stevens et al.*,*^31^ 2024	Prospective UK cohort; 734 lesions (86 MM/LM)	Histopathology	94.2	83.2	42.4	99.1	Real-world pathway; reduced number needed to treat (∼1.3); very high NPV.
Pizzichetta et al.*,*^70^ 2023	Multicenter; 55 amelanotic/hypomelanotic LM/LMM lesions	Histopathology	NR*	NR*	NR*	NR*	Feature-based multivariable model; modest accuracy gain but major increase in diagnostic confidence.
Gouveia et al.*,*^7^ 2023	Prospective; 229 LM/LMM (19 invasive LMM)	Histopathology	89†	90†	NR	NR	Detection of invasive component; predictive model AUC 74 %; moderate interobserver reproducibility (κ≈0.4).

NR = not reported. *Primary endpoint was feature-based modelling rather than standalone sensitivity/specificity. †Sensitivity and specificity refer to detection of invasive component within LM/LMM. Abbreviations: LM = lentigo maligna; LMM = lentigo maligna melanoma; MM = malignant melanoma; PPV = positive predictive value; NPV = negative predictive value; EF-LM = extra-facial LM.

**Differentiating benign macules from LM**. First, Guitera et al. developed an RCM scoring algorithm to differentiate LM from benign facial macules.^27^ In a retrospective series of 81 LMs and 203 benign macules, two major features (non-edged dermal papillae; large round pagetoid cells > 20 µm) and four minor features (≥3 atypical junctional cells in five 0.5 × 0.5 mm² fields; follicular localization of atypical cells; nucleated cells in dermal papillae; minus one point for a broadened honeycomb pattern) were combined. A threshold score ≥2 yielded 85 % sensitivity and 76 % specificity for LM (OR 18.6; 95 % CI 9.3–37.1) with 87 % interobserver reproducibility. In an independent test set (29 LMs and 44 benign macules), sensitivity and specificity were 93 % and 82 %, respectively. In a prospective handheld-RCM study,^28^ Menge et al. explicitly applied the same diagnostic algorithm,^27^ achieving 100 % sensitivity and 71 % specificity (85 % PPV, 100 % NPV) across 63 equivocal sites, although false positives were mainly related to actinically damaged skin. These findings illustrate how sensitivity remains high across settings, whereas specificity is more dependent on background photodamage and benign mimickers*.* In a meta-analysis by Nie *et al*., including 479 patients from six studies, RCM demonstrated a significantly higher specificity (mean difference [MD] 19.10, 95 % CI 0.93–37.28, *p* = 0.04) and a trend toward increased sensitivity (MD 14.56, 95 % CI 0.29–28.83, *p* = 0.05) compared to dermoscopy for the diagnosis of LM.^29^

**Distinguishing LM from early LMM**. In complex cases, LM may require a more conservative approach, and non-surgical options can be considered. When such strategies are being explored—particularly when complete excision was not achieved during initial surgery—ruling out dermal invasion becomes essential, as distinguishing LM from LMM is critical for guiding appropriate management. Several studies have explored the added value of RCM in identifying early dermal invasion. In a prospective case–control study of 229 histologically confirmed LM/LMM lesions, Gouveia *et al*. evaluated the ability of RCM to detect invasion and found that it correctly identified dermal invasion in 89 % of LMMs and excluded it in 90 % of LMs.^7^ A multivariate model based on epidermal disarray, large melanocytes, and nests achieved moderate diagnostic accuracy, supporting the role of RCM in detecting early invasion.^7^ In a retrospective multicentre analysis of 180 lesions, Guida *et al*. further confirmed the association between dermal invasion and specific RCM features, such as medusa head-like structures, dermal nests, and nucleated cells in dermal papillae.^30^ Dermal nests were observed exclusively in invasive lesions. This study also highlighted correlations between dermoscopic and confocal features, supporting the integrated use of both modalities to guide diagnostic and surgical decisions.

**Expanding to extra-facial LM**. Licata et al. addressed the challenge of diagnosing extra-facial LM (EF-LM), which often lacks classical dermoscopic patterns.^26^ In a multicentre case–control study including 48 EF-LM and 45 benign lesions, RCM achieved 90 % sensitivity and 97 % specificity. EF-LM exhibited significantly higher rates of pagetoid spread, dendritic and round cells in the epidermis, atypical junctional cells, and a meshwork pattern compared to benign mimickers.

**Evidence from real-world clinical practice**. In a large prospective UK cohort (734 lesions), Stevens et al.^31^ confirmed high sensitivity (94.2 %) and particularly high NPV (99.1 %), supporting RCM as a reliable triage tool in routine practice. Importantly, diagnostic performance varied by lesion type and clinical scenario, reinforcing the need for stratified interpretation rather than pooled estimates.

Overall, accumulating evidence demonstrates that RCM consistently improves diagnostic sensitivity across LM/LMM presentations, while specificity and predictive values vary according to lesion characteristics and clinical indication, underscoring the importance of contextualized interpretation as summarized in Table 4.

### RCM for preoperative margin mapping

Presurgical RCM mapping emerged as an effective strategy to identify subclinical extension, with several studies demonstrating clinical benefit (Table 3); however, reported outcomes vary substantially according to mapping strategy, surgical workflow, study design, and chosen endpoints (Table 5). In the earliest Australian series, Collgros *et al*. applied arm‑mounted RCM to 15 of 102 LM/LMM lesions and saw recurrences drop from 38 % overall to 27 % in the RCM‑mapped group—a nearly 30 % relative reduction—even among 48 recurrent tumours (33 % re‑recurrence).^32^ Guitera and colleagues built on this by generating four‑directional mosaics and targeted 2–3 mm punch biopsies in 37 high‑risk lesions^33^; 59 % of tumours extended >5 mm past their clinical borders, and RCM findings prompted wider excisions or alternate therapies in 73 % of cases. Importantly, included studies address different clinical objectives, ranging from margin concordance and first-stage clearance to recurrence prevention and workflow optimisation, precluding direct quantitative pooling.

**Table 5:** RCM-assisted margin delineation in LM/LMM prior to excision: key evidence.

Study	Design / n	RCM approach	Key quantitative outcomes	Clinical impact / nuance
Champin et al.,^34^ 2014	Prospective; *n* = 33 LM	VivaScope + spaghetti technique	85 % first-pass negativity; mean 1.2 strips; mean final margin 2.7 mm; 0 recurrences (mean FU 10 mo)	RCM-guided spaghetti enabled tissue-sparing excision with minimal re-intervention; residual LM <5 % of first strip length in failures
Yélamos et al.*,*^53^ 2017	Prospective; 22 pts / 23 LM/LMM	Handheld RCM + radial video mosaicking	RCM margins only 0.76 mm smaller than histology; estimated defect 6.34 cm² vs. final 7.74 cm²	High spatial concordance with staged excision; RCM mapping helped anticipate defect size and spare healthy tissue
Couty et al.*,*^55^ 2018	Prospective; *n* = 70 LM/LMM	RCM + spaghetti technique	Mean 1.13 strips; 0 recurrences at 44 months; 88 % tumor-free first strip	Confirmed Champin results in larger cohort with durable control and excellent cosmetic outcomes
Cinotti et al.*,*^40^ 2020	Prospective; *n* = 42 LM/LMM (+2 mucosal)	In vivo RCM circumferential mapping ± 5 mm + ex vivo confocal microscopy (EVCM)	Correct margin classification 97.6 % (RCM) and 95.5 % (EVCM); 1 FN, 1 FP; 1 recurrence at ∼5 years	Proof‑of‑concept hybrid workflow validating high concordance with histology; not comparative; reconstruction delayed pending margin assessment
Collgros et al.*,*^32^ 2021	Retrospective; *n* = 117 LM/LMM lesions	Pre‑operative RCM mapping vs. standard excision	Recurrence: 27 % with RCM mapping vs. 38 % without RCM	Suggests reduced long‑term recurrence with RCM guidance despite heterogeneous designs and follow‑up
Gao et al.*,*^41^ 2020	Retrospective; *n* = 47 LM	Handheld RCM presurgical mapping	Mean reconstruction delay reduced (14.6 days mapped vs. 27.0 days non‑mapped)	RCM mapping facilitated faster reconstruction planning; workflow benefit rather than margin clearance endpoint
Navarrete‑Dechent et al..,^51^ 2023	Prospective; *n* = 72 LM/LMM	Handheld RCM quadrant mapping prior to staged excision	Residual tumor detection: Sens 96.7 %, Spec 66.7 %; overall margin agreement 85.9 % (κ = 0.71)	Large prospective validation with quadrant-by-quadrant histology; shows strong correlation but moderate specificity
Elshot et al.*,*^52^ 2021	Retrospective; *n* = 26 LM/LMM	Handheld RCM circumferential mapping with adhesive rings	Subclinical LM detected in 45.8 %; Sens 90 %, Spec 86 %; negative margins in 95.8 %; recurrence 4.8 %	Demonstrates feasibility of HH‑RCM-guided conventional excision with low overtreatment
Cabrioli et al..,^39^ 2023	Retrospective; *n* = 57 LM/LMM	RCM vs. dermoscopy margin mapping	RCM: Sens 94.7 %, Spec 83.3 %, PPV 94.7 %, NPV 83.3 %	RCM reduced re-interventions and overtreatment, especially in facial lesions
Mathieu et al.*,*^37^ 2025	Retrospective cohort; *n* = 282 (184 RCM vs. 98 controls)	Presurgical RCM mapping vs. standard excision	First-stage clearance 63.6 % vs. 34.7 %; mean stages 1.18 vs. 1.94; margins 5.2 vs. 6.2 mm	Largest comparative cohort showing improved efficiency and tissue preservation with RCM

Abbreviations: LM = lentigo maligna; LMM = lentigo maligna melanoma; RCM = reflectance confocal microscopy; FN = false negative; FP = false positive. All outcomes are reported against histopathology following staged or conventional excision.

The switch to handheld probes (VivaScope 3000®) accelerated adoption: Champin *et al*. guided narrow, 2 mm “spaghetti” strips in 33 facial LM patients, achieving an 85 % first‑pass negativity with just 1.2 strips on average and no recurrences at 10 months.^34^ Couty et al. confirmed these results in 70 consecutive cases,^35^ reducing the average number of peripheral strips to 1.13 (versus 1.55–2.21 historically) and reporting zero local recurrences over 44 months. Around the same time, Pellacani’s SMART protocol^36^ demonstrated that shallow skin cuts could serve as reproducible landmarks: of 126 margin checks, 26 % were clear at first step, 48 % at second, and 17 % at third, all histologically confirmed, with fair interobserver agreement (κ≈0.43). These early prospective series were conducted in expert single-center settings using standardized “spaghetti” or landmark-based protocols, which may not be directly generalizable to broader clinical practice.

Most recently, our group reported the largest single-center retrospective cohort to date, including 282 LM/LMM patients.^37^ Preoperative RCM mapping significantly increased the rate of complete excision at first stage (63.6 % vs. 34.7 %, *p* < 0.0001), reduced the mean number of surgical stages (1.18 vs. 1.94, *p* < 0.0001), and allowed narrower margins (5.2 vs. 6.2 mm, *p* = 0.002) compared to standard care. The advantage of RCM was especially pronounced in surgically complex facial regions (cheek, temporal area, eyelid), underscoring its role in optimizing oncological control while preserving tissue.

Given this methodological variability, higher-level syntheses are helpful to contextualize individual series. Two systematic syntheses have since distilled these data into concise performance metrics: Elshot et al.’s 2023 review^10^ (5059 LM, 1271 LMM) found that RCM mapping before wide local excision cut local recurrence to 2 % (2/84) vs. 13 % after standard 5 mm margins, and trimmed the average number of surgical stages from 1.7 to 1.1 (*p* < 0.0001), while Mesquita et al.’s 2024 meta‑analysis^38^ of nine RCM mapping cohorts (329 participants) reported an RCM–histology concordance of 92.1 %, a negative predictive value of 89.2 %, pooled sensitivity and specificity of 91.4 % and 95.7 %, and a mean of just 1.16 surgical stages per lesion.

Moreover, Melhoranse Gouveia et al.’s 2024 meta‑analysis^23^ of 303 LM/LMM lesions identified key RCM features that reliably mark borders—non‑edged dermal papillae (OR 4.50), widespread pagetoid cells (OR 25.06), dermo‑epidermal junction disarray (OR 4.19) and junctional nests (OR 4.48)—further guiding precise margin delineation.

**Figure 3:**
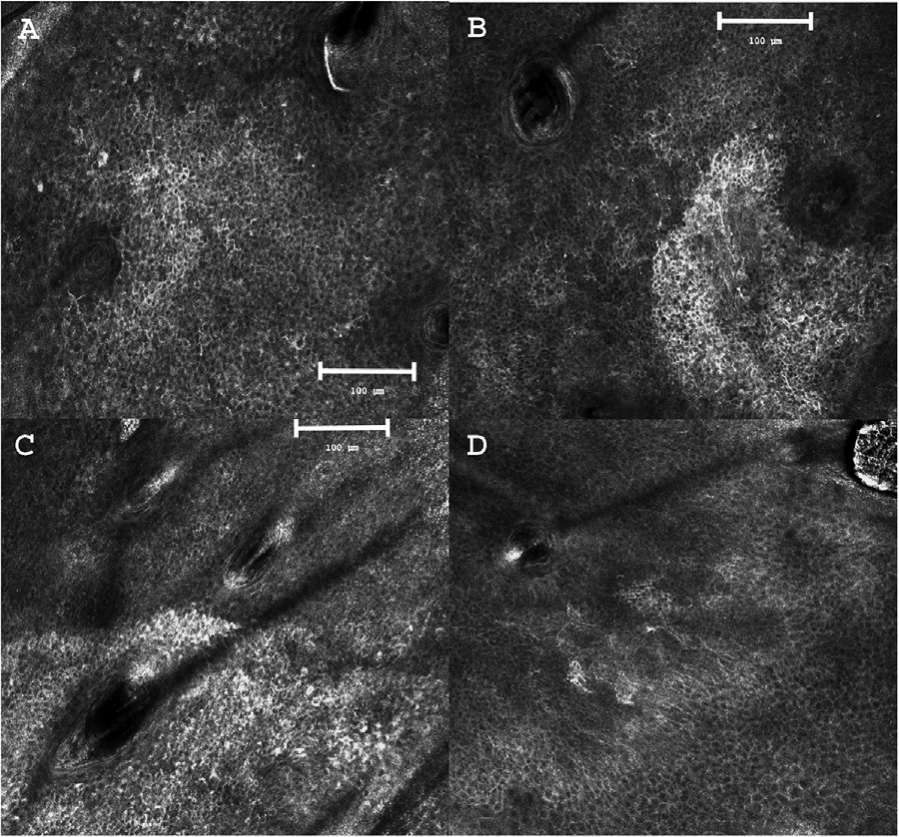
Reflectance confocal microscopy findings of lentigo maligna during preoperative margin mapping. (A) “Spider web”–like appearance with dendritic hyperreflective cells surrounding a hair follicle, a characteristic feature of lentigo maligna. (B) Abrupt transition zone with asymmetric hyperreflectivity: marked architectural disorganization on the right side of the follicle and less intense but equally atypical changes on the left side, both consistent with lentigo maligna. (C) RCM-defined margin showing clusters of atypical lentigo maligna cells around a follicular structure. (D) Adjacent field located only a few millimeters from panel C, demonstrating persistent architectural disarray and dendritic cells extending into deeper layers, further highlighting the subclinical spread of the lesion. Images adapted from Mathieu et al.^37^ These panels are provided as representative illustrative examples of commonly described RCM patterns in lentigo maligna and margin mapping.

Altogether, despite substantial heterogeneity in mapping protocols and outcome measures, presurgical RCM mapping consistently improves margin delineation, reduces re-interventions, and supports tissue preservation across diverse clinical settings, as summarized in Table 5. Representative RCM images illustrate abrupt transition zones and subclinical extension beyond clinical borders (Figure 3).

### Impact of RCM on surgical strategy

RCM has fundamentally shifted surgical management of LM and LMM by enabling:

**Personalized margin planning**. In vivo mapping defines lesion boundaries at a cellular level, allowing surgeons to tailor excision margins precisely rather than relying on empiric wide margins. Studies have shown that RCM-guided margins can be up to 60 % larger than clinical estimates, reducing the risk of residual disease while avoiding unnecessary removal of healthy tissue.^33^

**Reduced number of surgical stages**. By integrating RCM into staged excision and Mohs workflows, centers report a decrease in the average number of stages needed to achieve clear margins^37^, ^39^—from 1.7 to 1.1 in RCM-mapped cases—and improvements in first-stage clearance from 50 % to 86 %.^10^

**Enhanced tissue conservation**. Especially in cosmetically sensitive areas (face, neck, hands), RCM mapping spares normal skin by precisely delimiting subclinical spread.^39^ Techniques such as the “spaghetti” method and SMART approach combine RCM guidance with minimal peripheral sampling to maintain aesthetic outcomes.^34^, ^35^, ^36^

**Real-time intraoperative decision-making**. *Ex vivo* confocal microscopy complements in vivo RCM by verifying margins immediately after excision, enabling same-session re-excisions when needed and reducing delays and patient anxiety associated with delayed histopathology.^40^

**Optimized reconstruction timing.** Faster confirmation of clear margins expedites definitive closure or reconstructive procedures by shaving days off the interval between excision and repair (e.g., mean time-to-repair reduced from 27.0 to 14.6 days).^41^

**Potential to guide non-surgical therapies**. In patients who are poor surgical candidates or who opt for non-surgical management, RCM provides a valuable non-invasive tool to guide treatment selection, monitor therapeutic response, and detect early persistence or recurrence.

Guitera et al. demonstrated that RCM significantly outperformed dermoscopy in identifying treatment failure after non-surgical therapy,^42^ with an LM score achieving 100 % sensitivity and 94 % specificity for recurrent LM, compared with 80 % sensitivity and 56 % specificity for dermoscopy—even when performed by expert clinicians. A key advantage of RCM was its ability to distinguish residual atypical melanocytes from melanophages and post-treatment inflammatory changes.

Similarly, Alarcon et al. prospectively evaluated LM patients treated with imiquimod and showed that RCM accurately identified all true non-responders, with diagnostic performance comparable to histopathology and no false-negative results.^43^ Importantly, RCM enabled early recognition of persistent pagetoid or junctional atypia before clinical or dermoscopic recurrence became apparent, facilitating timely conversion to surgical management when needed.

**Surveillance after surgical treatment.** RCM plays a key role in evaluating repigmentation within or adjacent to surgical scars—one of the most challenging diagnostic scenarios due to post-inflammatory changes and loss of follicular landmarks. Navarrete-Dechent et al. reported that RCM achieved 95 % sensitivity and 78 % specificity for detecting recurrent LM/LMM in scars, outperforming dermoscopy, which often showed only nonspecific homogeneous brown pigmentation.^44^ Importantly, RCM identified hallmark features of recurrence such as bright nucleated cells at the dermoepidermal junction and folliculotropism, enabling accurate distinction from benign causes like actinic keratosis or postinflammatory hyperpigmentation. These capabilities allow clinicians to select the most suspicious biopsy sites, reduce sampling error, and improve early detection of recurrence—even years after initial surgery.

From a health-system perspective, decreased reintervention rates and streamlined surgical pathways may partially offset the higher upfront costs of RCM by reducing operative time, pathology workload, and long-term follow-up associated with local recurrence.

## Discussion

This systematic review highlights a consistent signal in favor of RCM across both diagnostic assessment and presurgical management of LM/LMM. Despite heterogeneous study designs, RCM demonstrated high sensitivity for LM/LMM detection and was repeatedly associated with improved first-stage clearance, fewer surgical stages, narrower margins, and reduced local recurrence when integrated into presurgical workflows.

However, the strength of evidence is limited by substantial methodological heterogeneity, including variability in patient populations, clinical objectives (diagnosis, invasion assessment, margin mapping), RCM protocols, and reported endpoints. While diagnostic sensitivity was consistently high, specificity and predictive values varied according to lesion subtype, background photodamage, reader expertise, and clinical indication. Similarly, mapping outcomes depended strongly on mapping strategy and surgical workflow. These sources of variability are summarized in 4 and 5. Taken together, available evidence supports RCM as a valuable decision-support tool in specialized centers, but underscores the need for standardized protocols, multicenter validation, and prospective comparative trials before broader implementation.

### Limitations and practical challenges

**Access & Cost**. Access and cost remain major barriers to the widespread implementation of RCM. Commercial systems (including VivaScope 1500® and 3000® platforms) require substantial upfront investment, typically reported in the range of several tens to over one hundred thousand euros, with additional maintenance costs; per-examination expenses vary according to local organization, duration, and expertise. Robust LM/LMM-specific cost-effectiveness analyses are currently lacking, limiting firm economic conclusions. Nevertheless, multiple studies suggest that RCM-guided margin mapping may reduce the number of surgical stages, allow narrower excision margins, and lower local recurrence, potentially offsetting higher initial costs by decreasing operative time, re-excisions, and long-term follow-up burden.

Beyond RCM, optical coherence tomography (OCT) and multiphoton microscopy (MPM) are increasingly discussed as non-invasive alternatives.^45^, ^46^, ^47^, ^48^ OCT provides greater penetration depth (up to ∼1 mm) at acquisition and per-examination costs generally comparable to or slightly lower than RCM, but evidence for LM/LMM margin mapping remains less mature, supporting a complementary rather than substitutive role. In contrast, MPM remains largely confined to research settings due to substantially higher acquisition and maintenance costs and the absence of established reimbursement pathways. Overall, while direct head-to-head economic comparisons are scarce, the current balance of clinical evidence and implementation maturity supports RCM as the most established investment for LM/LMM margin mapping in specialized centers.

**Learning Curve & Variability.** Accurate image interpretation demands specialized training. Subtle features (e.g., pagetoid cells vs. Langerhans cells) can be confused,^49^^,^^50^ yielding only moderate interobserver agreement (κ ≈ 0.4–0.7).^36^ In routine practice, this variability may limit reproducibility across centers and reduce confidence among less experienced users, particularly in borderline or subtle cases. However, several factors are likely to mitigate this limitation. Structured training programmes, use of validated scoring systems, and consensus-based interpretation criteria have been shown to improve agreement. In addition, the increasing integration of artificial intelligence–assisted image analysis may further enhance reproducibility by standardizing feature detection and reducing operator dependence. Together, these developments support the feasibility of broader clinical adoption of RCM despite moderate interobserver variability.

**Depth & Field-of-View Constraints.** RCM penetrates only the superficial dermis (≤300 µm) and captures small snapshots (0.5–0.75 mm²).^14^ Comprehensive margin mapping requires time-intensive mosaicking or quadrant-based protocols, which can be cumbersome in busy clinics.

**Workflow Integration.** Coordinating in vivo imaging before surgery and *ex vivo* RCM after excision adds procedural steps^40^ and can be very time-consuming—mapping a single lesion often requires 20–60 min.^51^ First, the clinician determines the visible lesion border by clinical and dermoscopic assessment. The VivaScope probe is then placed directly on the skin—either the arm‑mounted VivaScope 1500® for large‑area mosaics (up to 8 × 8 mm²) or the handheld VivaScope 3000® for flexible point‑by‑point imaging in concave or mobile sites.

Several orientation strategies have been described (Table 6):

• Paper tape or adhesive rings^39^^,^^51^, ^52^, ^53^, ^54^: tracing the clinical margin with tape or rings and sequentially imaging along its edge, shifting outward until no residual abnormalities are seen
• Shallow skin cuts (SMART approach)^36^: superficial cuts at predefined points serve as fixed landmarks to guide radial RCM imaging until negative margins are reached.
• Indentation mapping^34^^,^^55^: using the handheld probe’s footprint to create successive reference points for outward imaging.
• Four‑directional radial exploration.^33^ Sequential mosaics along four axes from lesion center until LM features are no longer observed.

**Table 6:** Comparison of presurgical RCM margin-mapping techniques in lentigo maligna and lentigo maligna melanoma.

Technique	Principle	Advantages	Limitations	Typical settings
Paper tape/adhesive rings^39^	Clinical margin is marked with tape or rings; sequential RCM imaging performed along and beyond the marked border until no LM features are detected	Simple, inexpensive, widely available; intuitive orientation	Manual, time-consuming; limited reproducibility; operator-dependent	Routine clinical practice, facial LM
SMART approach^36^	Shallow skin cuts used as fixed landmarks for radial RCM exploration	Reproducible landmarks; structured workflow	Minimally invasive; learning curve	High-risk LM, expert centers
Indentation mapping^34^^,^^55^	Handheld probe footprint used as a reference for stepwise outward imaging	Non-invasive; flexible; suitable for curved areas	Orientation less precise; limited standardization	Concave or mobile anatomical sites
Four-directional radial exploration^33^	Systematic RCM mosaics acquired along four axes from lesion center	Structured and reproducible approach	May miss asymmetric subclinical spread	Well-defined lesions

Across these modalities, the process consistently involves clinical/dermoscopic identification, placement of landmarks, systematic RCM imaging, and iterative outward adjustment until no confocal signs of LM/LMM remain. Despite their variety, these techniques remain largely artisanal and center specific. No standardized, guideline-endorsed protocol currently exists, underscoring the need for reproducible and efficient workflows.

Finally, to preserve the validity of RCM findings and ensure accurate excision, the time between RCM mapping and surgical removal should be minimized, ideally performing both on the same day within a coordinated day‑hospital schedule.

**Patient & Anatomic Factors.** Imaging curved or mobile sites (periocular, nasal ala, retroauricular) remains challenging—even with handheld probes.^39^^,^^56^ Long acquisitions can cause patient discomfort and motion artifacts that degrade image quality.

**Reimbursement & Standardization.** Few health systems reimburse RCM procedures, creating financial barriers for clinics. Importantly, the clinical experience summarized in this review largely originates from a limited number of early-adopting countries and centers, including the United States, Australia, and parts of Western Europe, and therefore reflects implementation models from these pioneering settings. By contrast, in many healthcare systems RCM remains limited by lack of reimbursement, high upfront costs, and absence of structured training programmes. Common enabling factors across countries appear to include centralization of care in specialized centers, multidisciplinary workflows, and access to formal training. Robust cross-country comparative implementation data remain lacking, representing an important area for future health-services research. Mapping techniques and reporting formats also vary widely, complicating multicenter studies and consensus guideline development.

### Future perspectives and clinical integration

In the coming years, the integration of machine-learning–based image analysis into clinical RCM devices may improve workflow efficiency and reproducibility in LM/LMM management.^57^ Proof-of-concept studies have reported promising performance for automated layer segmentation and dermal–epidermal junction delineation,^58^^,^^59^ as well as high accuracy for classifying LM versus benign macules using deep-learning architectures.^60^, ^61^, ^62^

To enable routine clinical adoption, several prerequisites remain: optimized image acquisition and contrast to support robust feature detection,^57^ large multi-center annotated datasets spanning phototypes and anatomical sites,^63^ and consensus standards for annotation, validation metrics, and decision thresholds. RCM’s near-histological, non-invasive imaging is also being explored in other dermatologic and cosmetologic indications.^64^ Ultimately, validated AI-assisted interpretation could support more standardized margin assessment and reporting, alongside training and certification pathways, reimbursement strategies, and prospective studies.

This review is limited by the heterogeneity of study designs, variable reporting of outcomes, and moderate methodological quality in several included studies. The absence of standardized RCM protocols and the lack of large randomized trials restrict the generalizability of findings. In addition, our literature search was restricted to three major academic databases and did not systematically include conference proceedings, clinical trial registries, or expert consultation, which may have led to underrepresentation of unpublished or negative studies and introduces a potential risk of publication bias. Overall, RCM improves diagnostic accuracy and preoperative margin delineation in LM/LMM, enabling tissue-sparing surgery and reliable surveillance. Wider adoption will depend on standardized mapping protocols, training and certification pathways, reimbursement, and further prospective studies integrating patient-reported and health-economic outcomes.

## Funding

None.

## Ethical approval

Not required.

## Data availability

The data that support the findings of this study are available from the corresponding author upon reasonable request.

## Author contribution

Olivier Mathieu: Conceptualization, Data curation, Formal analysis, Investigation, Methodology, Writing original draft, Douglas Henderson: Writing – review and editing, Visualization, Emilien Ezine: Writing – review and editing, Kevin Serror: Conceptualization, Methodology, Barouyr Baroudjian: Writing – review and editing, Maxime Battistella: Supervision, Validation, Writing – review and editing, Céleste Lebbé: Conceptualization, Supervision, Validation, Writing – review and editing, Marc Chaouat: Validation, Writing – review and editing, David Boccara: Supervision, Project administration, Validation, Writing – review and editing.

## Declaration of competing interest

None.

## Appendix Supplementary materials

Table S1 PRISMA check-list Image, application 1

Table S2 Methodological items for non-randomized studies (MINORS) instrument. Image, application 2

Table S3 Methodological items for systematic reviews and meta-analyses (AMSTAR-2) instrument. Image, application 3
