# Supplementary material for: Reflectance confocal microscopy in the management of lentigo maligna and lentigo maligna melanoma: a systematic review
Source: JPRAS Open. 2026 Mar 4;49:314–33. doi: 10.1016/j.jpra.2026.02.022 (PMC13019939; doi:10.1016/j.jpra.2026.02.022)
Supplement: Table S2 — Methodological items for non-randomized studies (MINORS) instrument. [file mmc2.docx]

**Methodological items for non-randomized studies (MINORS) instrument. Items**

**are scored as 0 (not reported), 1 (reported but inadequate), or 2 (reported and adequate).**

**The maximum score for non-comparative studies is 16, and for comparative studies is 24.**

(1) A clearly stated aim: The question addressed should be precise and relevant in the light of

available literature.

(2) Inclusion of consecutive patients: All patients potentially fir for inclusion (satisfying the

criteria for inclusion) have been included in the study during the study period

(3) Prospective collection of data: Data were collected according to a protocol established before

the beginning of the study.

(4) Endpoints appropriate to the aim of the study: Unambiguous explanation of the criteria used

to evaluate the main outcome, which should be in accordance with the question addressed by

the study. Also, the endpoints should be assessed on an intention-to-treat basis.

(5) Unbiased assessment of the study endpoint: Blind evaluation of objective endpoints and

double-blind evaluation of subjective endpoints. Otherwise the reasons for not blinding should

b stated.

(6) Follow-up period appropriate to the aim of the study: The follow-up should be sufficiently

long to allow the assessment of the main endpoint and possible adverse events.

(7) Loss to follow up less than 5%: All patient should be included in the follow up. Otherwise,

the proportion lost to follow up should not exceed the proportion experiencing the major

endpoint.

(8) Prospective calculation of study size: Information of the size of detectable difference of

interest with a calculation of 95% confidence interval, according to the expected incidence of

the outcome event, and information about the level for statistical significance and estimates of

power when comparing outcomes.

**Additional criteria in the case of comparative studies.**

(9) An adequate control group: Having a gold standard diagnostic test or therapeutic intervention

recognized as the optimal intervention according to the available published data.

(10) Contemporary groups: Control and studied group should be managed during the same time

period (no historical controls)

(11) Baseline equivalence of groups: The groups should be similar regarding the criteria other

than the studied endpoints. Absence of confounding factors that could bias the interpretation of

results.

(12) Adequate statistical analyses: Whether the statistics were in accordance with the type ofstudy with calculation of confidence intervals or relative risk.
